# The Role of IL-23 in the Development of Inflammatory Diseases

**DOI:** 10.3390/biology14040347

**Published:** 2025-03-27

**Authors:** Mario García-Domínguez

**Affiliations:** 1 Program of Immunology and Immunotherapy, CIMA-Universidad de Navarra, 31008 Pamplona, Spain; mgdom@unav.es; 2 Department of Immunology and Immunotherapy, Clínica Universidad de Navarra, 31008 Pamplona, Spain; 3 Centro de Investigación Biomédica en Red de Cáncer (CIBERONC), 28029 Madrid, Spain

**Keywords:** interleukin 23, inflammation, pain, inflammatory disease, immune system

## Abstract

Interleukin-23 is crucial in the initiation and progression of certain inflammatory disorders. As a key cytokine, IL-23 is involved in the differentiation and activation of Th17 cells, which play a role in a broad spectrum of inflammatory diseases. This review examines the molecular mechanisms through which IL-23 contributes to the pathogenesis of conditions including psoriasis, rheumatoid arthritis, inflammatory bowel disease, and multiple sclerosis. By elucidating the significant role of IL-23 in inflammation, this review underscores its importance as a therapeutic target for managing inflammatory conditions, with particular emphasis on current and emerging biologic treatments.

## Simple Summary

Interleukin-23 is a crucial protein produced by the immune system that plays a role in triggering and developing certain inflammatory diseases. It stimulates specific immune cells, like Th17 cells, which are involved in inflammatory diseases including psoriasis, rheumatoid arthritis, inflammatory bowel disease, and multiple sclerosis. This review examines how IL-23 contributes to these diseases and discusses the potential of treatments that target IL-23 and its receptor.

## 1. Introduction

Inflammation is a fundamental biological process that plays a critical role in immune defense and the maintenance of tissue homeostasis [1]. This highly regulated and dynamic response functions as a protective mechanism, enabling an organism to detect and eliminate harmful agents, such as pathogens, apoptotic and necrotic cells, and toxic substances [2]. Under physiological conditions, the inflammatory response is controlled to ensure effective immune function while minimizing tissue damage. Once the harmful stimulus is eliminated, resolution mechanisms actively restore homeostasis, preventing unnecessary tissue injury [3].

At the molecular level, inflammation is initiated by the activation of pattern recognition receptors (PRRs), such as Toll-like receptors (TLRs) and NOD-like receptors (NLRs), upon the recognition of pathogen-associated molecular patterns (PAMPs) and damage-associated molecular patterns (DAMPs) [4]. This activation triggers intracellular signaling cascades, such as the NF-κB transcription factor and MAPK pathways, which drive the transcription of key pro-inflammatory cytokines such as TNF-α, IL-1β, and IL-6 [5]. These cytokines further amplify the inflammatory response by recruiting immune cells, including neutrophils, macrophages, and lymphocytes, to the site of infection or injury [6].

The resolution of inflammation is an active and tightly coordinated process mediated by specialized pro-resolving lipid mediators (SPMs), including resolvins, protectins, and maresins, which are derived from ω-3 polyunsaturated fatty acids [7]. These biomolecules counteract pro-inflammatory signaling, evoke the macrophage-mediated clearance of apoptotic cells (efferocytosis), and inhibit excessive neutrophil infiltration, ultimately restoring tissue integrity and function [8]. The dysregulation of these pathways promotes sustained inflammation and contributes to the development of many chronic inflammatory diseases, in which the immune system erroneously attacks the body’s own tissues [9].

Prolonged and uncontrolled inflammation is strongly linked to several diseases that compromise quality of life [10]. Among these, chronic inflammatory disorders like psoriasis, inflammatory bowel disease (IBD), rheumatoid arthritis (RA), and multiple sclerosis (MS) are the most common and clinically significant manifestations of immune dysregulation [11,12,13]. In these pathological conditions, the dysregulated activation of immune cells, including Th cells, B cells, and antigen-presenting cells (APCs), results in the biosynthesis of pro-inflammatory cytokines such as IFN-γ, IL-17, and GM-CSF [14,15]. These cytokines play a key role in sustaining and amplifying the inflammatory response, creating a vicious cycle in which inflammation persists, leading to tissue damage [16]. Among them, IL-23 has emerged as a critical mediator in the pathogenesis of autoimmune and inflammatory diseases, attracting considerable scientific interest [17].

IL-23 is a heterodimeric cytokine composed of two distinct subunits: p19, which confers its specific biological function [18], and p40, a component it shares with interleukin-12 (IL-12) [19]. It is predominantly produced by several APCs, such as macrophages and dendritic cells, in response to microbial stimuli and other immune signals [20]. Since its discovery, IL-23 has been identified as a regulator of pathogenic inflammation, primarily due to its role in driving the differentiation, expansion, and sustained activation of Th17 cells [21]. Th17 cells secrete a range of pro-inflammatory cytokines, such as IL-17, IL-22, and GM-CSF, which collectively contribute to immune cell activation and subsequent tissue damage [22]. While Th17 cells can fulfill protective and homeostatic functions under certain physiological conditions [23], their dysregulated activation in the presence of IL-23 drives persistent inflammation and progressive tissue destruction.

Given its central role in sustaining chronic inflammation, IL-23 has emerged as a key target for therapeutic intervention. Monoclonal antibodies designed to selectively neutralize IL-23 have demonstrated high efficacy in the treatment of autoimmune diseases, especially psoriasis and IBD [24]. These immunotherapies include ustekinumab, which targets the shared p40 subunit of both IL-12 and IL-23 [25], as well as more selective p19 inhibitors such as guselkumab and risankizumab [26,27]. By disrupting the IL-23-mediated inflammatory cascade, these treatments have yielded many clinical benefits, leading to symptom relief and disease remission in affected individuals.

This review provides a comprehensive evaluation of IL-23 in the onset and progression of inflammatory diseases. By evaluating its immunological functions, role in disease pathogenesis, and therapeutic potential, this paper highlights IL-23 as both a biomarker and a target for therapeutic intervention in many chronic inflammatory diseases. Enhancing our understanding of IL-23 in immune regulation will not only expand our knowledge of the mechanisms underlying inflammatory diseases but also contribute to the creation of more effective and personalized treatment approaches.

## 2. Biology of IL-23

In this section, the molecular structure of IL-23 as well as its receptor (IL-23R) will be thoroughly analyzed. The structure of IL-23 will be examined, focusing on the molecular interactions (with IL-23R) that allow IL-23 to perform its biological functions. Moreover, this section will explore the cellular actions triggered by the binding of IL-23 with IL-23R. Upon receptor activation, many intracellular signaling pathways are initiated. These signaling events are crucial to the role of IL-23 in immune regulation, particularly in the differentiation of Th17 cells and the subsequent production of pro-inflammatory cytokines. The consequences of the IL-23/IL-23R interaction will be discussed in the context of immune responses and inflammation. Finally, the relevance of IL-23 and IL-23R in the development of autoimmune and inflammatory diseases will be addressed.

### 2.1. Molecular Structure of IL-23

IL-23 is a heterodimeric cytokine with a molecular weight of approximately 59 kDa, composed of 476 amino acids [19]. It consists of two subunits: IL-23p19 (p19), a subunit unique to IL-23, and IL-12p40 (p40), a subunit shared with IL-12 [18,19]. Beyond its structural similarities with IL-12, IL-23 shares both evolutionary and functional links with other cytokines in the IL-12 family, such as IL-27 and IL-35 [28]. While IL-12 and IL-23 predominantly drive pro-inflammatory immune responses, IL-27 and IL-35 are mainly linked to immunoregulatory functions, underscoring the complex interplay among these cytokines in the maintenance of immune homeostasis [29].

**Figure 1:**
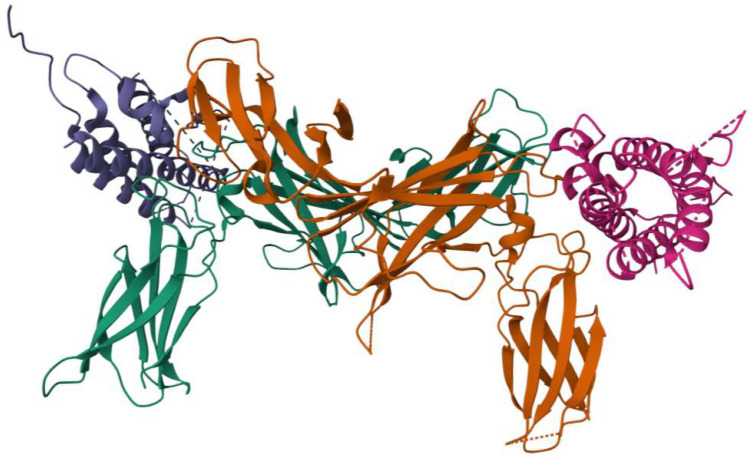
Three-dimensional structure of IL-23. Image provided by RCSB Protein Data Bank.

The crystal structure of IL-23 (Figure 1) provides important insights into its molecular architecture and functional interactions. IL-23p19 adopts a characteristic four-helix bundle cytokine fold, a structural motif commonly found in members of the IL-6 family, which facilitates its specific binding interactions [18]. On the other hand, IL-12p40 is organized into three distinct structural domains, forming a scaffold that plays a critical role in receptor engagement and signal transduction [19]. When the two subunits assemble, they form a complex three-dimensional structure that is essential to its biological function. The molecular configuration of IL-23 features a unique conformational arrangement, wherein a key arginine residue (Arg159) on helix D of IL-23p19 interacts with a highly specific binding site on IL-12p40, known as the “arginine pocket” [30]. This interaction is driven by numerous molecular forces, including hydrogen bonding, electrostatic interactions, and van der Waals forces, all of which contribute to the stability and receptor binding capacity of the cytokine [31].

Functionally, the p19 subunit is chiefly responsible for binding to the IL-23R complex, a pivotal determinant in the activation of downstream signaling pathways that promote Th17 cell differentiation and pro-inflammatory cytokine production [32]. In contrast, IL-12p40 is essential for mediating interactions between the IL-23 and the IL-12Rβ1, a receptor subunit also employed by IL-12 [31]. This dual-receptor binding characteristic emphasizes the evolutionary and functional relationship between IL-12 and IL-23, highlighting the different shared signaling mechanisms that regulate immune homeostasis and inflammatory responses [28].

### 2.2. IL-23 Receptor: Structure and Localization

The IL-23R complex is composed of two distinct type I transmembrane proteins: IL-12Rβ1 and IL-23R [31]. While IL-12Rβ1 serves as a shared receptor subunit also employed in the IL-12 receptor complex [33], IL-23R is unique to IL-23 signaling [34]. IL-23R is mainly expressed in several immune cells, playing a key role in mediating inflammatory responses and immune regulation [31]. Notably, its expression is found in macrophages [35], NKT cells [36], ILCs [37], γδ T cells [32], MAITs [38], and Th17 cells [32]. Structurally, IL-23R is a 629-amino acid glycoprotein with a molecular weight of approximately 70 kDa [39]. The receptor is composed of several functional domains, each contributing to its role in ligand binding and intracellular signal transduction. IL-23R contains an N-terminal signal peptide essential for its proper localization to the plasma membrane, a fibronectin type III-like domain that facilitates ligand binding and receptor dimerization, and an intracellular cytoplasmic tail enriched with three conserved tyrosine phosphorylation sites that serve as docking sites for downstream signaling molecules [39].

**Figure 2:**
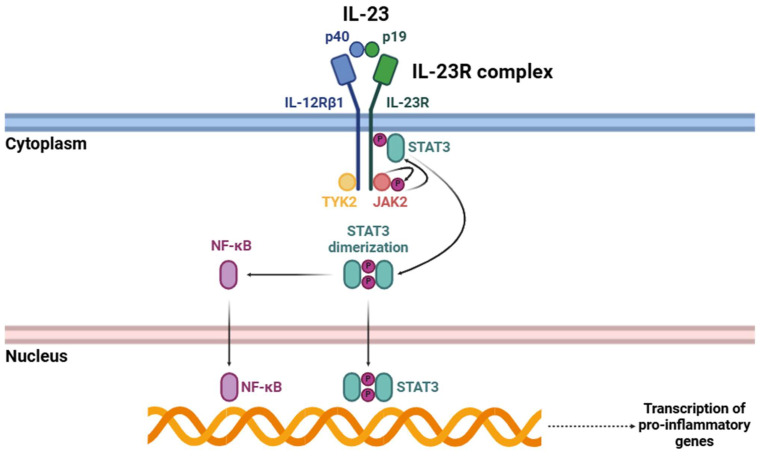
Signaling cascade triggered by the interaction of IL-23 with its receptor (IL-23R). Abbreviations: p40 (p40 subunit), p19 (p19 subunit), IL-12Rβ1 (interleukin 12 receptor β1), IL-23R (interleukin 23 receptor), STAT3 (signal transducer and activator of transcription 3), TYK2 (tyrosine kinase 2), JAK2 (Janus kinase 2), and NF-κB (nuclear factor kappa-light-chain-enhancer of activated B cells).

Upon IL-23 binding (Figure 2), IL-23R begins a complex intracellular signaling cascade mainly mediated by certain members of the JAK family [34]. IL-23R-associated JAK2 and IL-12Rβ1-associated TYK2 undergo activation through trans-phosphorylation events. These kinases subsequently phosphorylate specific tyrosine residues within the cytoplasmic domain of IL-23R, creating docking sites for STAT3 [24,40,41]. Phosphorylated STAT3 undergoes dimerization and subsequent translocation to the nucleus, where it acts as a transcriptional regulator, orchestrating the expression of many genes involved in immune activation [42]. Among these, one of the most critical targets is RORγt, a master transcription factor essential for Th17 cell differentiation [43].

Apart from the well-characterized JAK-STAT signaling pathway, the activation of IL-23R triggers other intracellular signaling pathways that further amplify its pro-inflammatory effects. One of the key pathways triggered by IL-23R is the NF-κB signaling pathway, which is crucial for regulating the transcription of numerous pro-inflammatory cytokines and other immune effector molecules [44,45]. Upon IL-23R activation, structural changes occur that facilitate the recruitment and activation of different intracellular signaling components. This process activates key kinases, including IκB kinase (IKK), which phosphorylates IκB proteins, leading to their degradation [46]. This proteolytic degradation releases NF-κB dimers, primarily p65/p50, allowing their translocation into the nucleus [47]. Once in the nucleus, these NF-κB dimers bind to specific DNA sequences within the promoters of pro-inflammatory genes, thereby triggering their transcription [48]. Consequently, this pathway enhances the production of various cytokines and effector molecules that contribute to the amplification and persistence of inflammatory responses.

Pro-inflammatory cytokines upregulated through the NF-κB signaling pathway include IL-6 [49], IL-17 [50], IL-21 [51], IL-22 [52], GM-CSF [53], and TNF-α [54]. These cytokines play a key role in inflammation by recruiting immune cells, as well as contributing to dysregulated tissue remodeling and maladaptive immune responses linked to chronic inflammatory diseases.

### 2.3. Biological Roles of IL-23

IL-23 plays a pivotal role in various immune responses, particularly in the regulation of inflammation and host defense mechanisms. This cytokine is synthesized in response to inflammatory stimuli, microbial invasion, or pathogenic insults, which trigger its production by various immune and non-immune cell types [55,56,57]. Among the most significant producers of IL-23 are antigen-presenting cells (APCs), including macrophages, monocytes, and dendritic cells (DCs) [58]. These cells respond to pathogen-associated molecular patterns (PAMPs) via pattern recognition receptors (PRRs) such as Toll-like receptors (TLRs), inducing the transcription and secretion of IL-23 [59].

Beyond these primary sources, IL-23 can also be synthesized by innate immune cells like neutrophils and innate lymphoid cells (ILCs), especially ILC3s. Neutrophils, recognized for their role in acute inflammation and pathogen clearance, have been found to contribute to the IL-23-mediated immune response under certain inflammatory conditions [60]. Similarly, ILC3s participate in mucosal immunity by responding to IL-23 signaling, leading to the production of IL-17 and IL-22, cytokines essential for epithelial barrier integrity and antimicrobial defense [61].

In addition to microbial stimuli, IL-23 production is influenced by various environmental and intracellular factors. Hypoxia and metabolic signals have been shown to modulate IL-23 expression, adding another layer of complexity to its regulation [62,63]. Hypoxic conditions, often present in inflamed tissues, enhance IL-23 synthesis via HIF-1α, further amplifying inflammatory responses [62]. Similarly, metabolic signals, such as disruptions in lipid metabolism within macrophages, can drive IL-23 production, linking immune activation to metabolic pathways [63].

**Figure 3:**
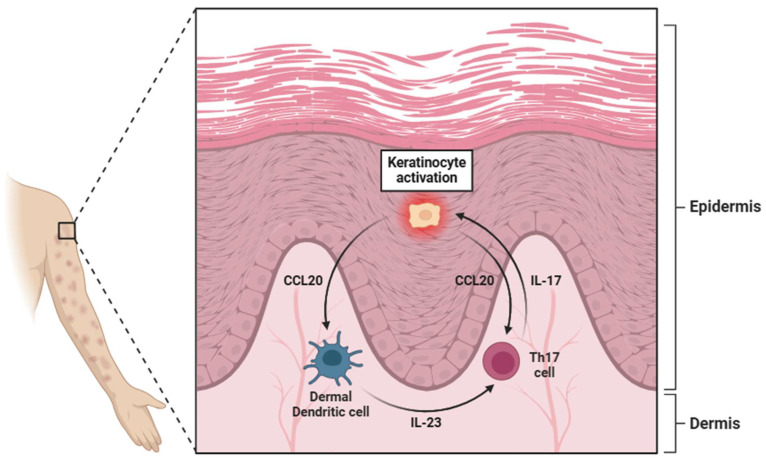
A diagram illustrating the pivotal role of keratinocytes in psoriasis. These cells activate dermal dendritic cells (dDCs) through the secretion of CCL20, which, through interaction with specific receptors (CCR6), stimulates the dDCs to release IL-23. This cytokine activates Th17 lymphocytes through binding to the IL-23R receptor. Once activated, Th17 cells secrete IL-17, which in turn further activates keratinocytes, reinforcing a positive feedback loop. However, the secretion of CCL20 by keratinocytes also exerts a degree of regulation over Th17 lymphocytes. Abbreviations: CCL20 (C-C motif chemokine ligand 20), IL-23 (interleukin 23), Th17 (T helper 17 cell), and IL-17 (interleukin 17).

In the skin, IL-23 is produced by a range of cell types, including keratinocytes, dermal dendritic cells (dDCs), and epidermal Langerhans cells (Figure 3) [64,65,66]. The cutaneous production of IL-23 is particularly relevant in the context of autoimmune and inflammatory skin disorders, such as psoriasis, where its overexpression contributes to chronic inflammation by promoting the expansion of Th17 cells [21]. Additionally, the gastrointestinal tract serves as another critical site of IL-23 synthesis, where its production is largely driven by Paneth cells (specialized epithelial cells located in the small intestinal crypts) [67]. These cells play an essential role in maintaining mucosal immunity and regulating the intestinal microbiota by releasing antimicrobial peptides in response to bacterial stimuli [68]. Additionally, within the gut-associated lymphoid tissue (GALT), DCs and macrophages produce IL-23, particularly in IBD [69,70].

The principal biological function of IL-23 lies in its crucial role in sustaining and amplifying the Th17 cell lineage, a subset of CD4^+^ T cells known for their potent pro-inflammatory activity [71]. Although IL-23 is not directly involved in the initial differentiation of naïve CD4^+^ T cells into Th17 cells (a process primarily driven by cytokines such as TGF-β and IL-6), it plays an indispensable role in the stabilization, expansion, and maintenance of this population [72,73]. Once Th17 cells have differentiated, IL-23 promotes their survival and proliferation, reinforcing their effector functions and enhancing their capacity to secrete pro-inflammatory cytokines, including IL-17, IL-21, and IL-22 [74]. These cytokines orchestrate a robust inflammatory cascade by activating immune cells at sites of infection or tissue damage [75].

Finally, accumulating evidence suggests that IL-23 plays a multifaceted role in tumor immunity, exerting pro-tumorigenic and anti-tumorigenic effects depending on the tumor microenvironment, immune cell composition, and cytokine signaling dynamics [76]. IL-23 promotes the recruitment and activation of numerous immune effector cells, including CD8^+^ T cells, NK cells, and neutrophils, within the TME [77]. These immune cells contribute to tumor cell lysis through various mechanisms: (1) IL-23 stimulates Th17 cells to produce IL-17A and IL-22, which enhance the infiltration of effector immune cells into the TME. IL-17A induces the expression of chemokines such as CCL20, CXCL1, and CXCL9, which facilitate the recruitment of DCs, NK cells, and CD8^+^ T lymphocytes [78,79]. (2) IL-23 promotes the maturation of DCs, enhancing their ability to present tumor antigens to naïve T cells, a crucial process for initiating adaptive immune responses that target tumor cells [80]. (3) IL-23 amplifies antitumor immunity by enhancing the cytotoxic activity of CD8^+^ T lymphocytes and NK cells, leading to increased production and secretion of perforin and granzyme B, which facilitate tumor cell apoptosis [81].

Paradoxically, IL-23 can also contribute to tumor progression in certain malignancies by creating a pro-inflammatory microenvironment that promotes immune evasion, angiogenesis, and malignant cell survival. This dual function is context-dependent and shaped by the presence of different immunosuppressive cells, cytokine interactions, and metabolic factors within the TME [82]. IL-23, through the action of IL-17, facilitates the recruitment of additional pro-inflammatory cells, including neutrophils and MDSCs [83]. These recruited cells have the ability to inhibit anti-tumor immunity, thereby enhancing the tumor’s capacity to evade immunosurveillance [84]. Simultaneously, IL-23 is implicated in the modulation of angiogenesis, a hallmark of tumor progression [85]. Through the induction of VEGF production (via IL-17), IL-23 indirectly facilitates neovascularization within the tumor, thereby ensuring an adequate supply of nutrients and oxygen to rapidly proliferating neoplastic cells [86]. This angiogenic response not only promotes tumor growth but also plays a role in creating a more intricate and immunosuppressive TME [87].

The impact of IL-23 on immune evasion is further reinforced by its interactions with Tregs. In numerous malignancies, IL-23 can enhance the accumulation of Tregs, which are known to exert immunosuppressive effects within the TME [88]. By increasing the expression of immunosuppressive molecules such as TGF-β and IL-10 [89,90], Tregs induced by IL-23 help to attenuate the activity of CTLs and NKs, thereby facilitating tumor immune escape [91].

These conditions can amplify the pro-tumorigenic effects of IL-23 by driving a metabolic shift that supports tumor cell survival and immune tolerance, thereby establishing a feedback loop that perpetuates the inflammatory microenvironment, which in turn promotes tumor growth.

## 3. Role of IL-23 in Pain-Associated Inflammatory Mechanisms

One of the most compelling pieces of evidence for the role of IL-23 in inflammatory pain comes from several studies involving genetically modified mice deficient in the IL-23p19 subunit, which is essential for the functional activity of IL-23 [92,93,94,95,96,97]. These studies have demonstrated that the absence of IL-23 exerts a profound protective effect against the development of zymosan-induced arthritis (ZIA), an established preclinical model of RA characterized by robust immune activation and pain-related behaviors [98]. Notably, IL-23p19-deficient mice exhibit a complete abolition of both the pathological hallmarks of ZIA and the behavioral manifestations of pain typically observed in this preclinical model, suggesting that IL-23 is indispensable for the initiation and propagation of inflammation-driven nociceptive signaling [93]. The absence of IL-23, as demonstrated in IL23p19^−/−^ mice, disrupts this intricate network, leading to a marked reduction in the recruitment and activation of immune cells within affected tissues, thereby mitigating pain hypersensitivity and tissue damage [94].

In the context of experimental autoimmune encephalomyelitis (EAE), the most commonly used experimental model for human inflammatory demyelinating disease, including MS [99], IL-23 stimulates myelin-reactive T cells to produce both IFN-γ and IL-17, contributing to the generation of encephalitogenic Th17 cells [100]. The differentiation of naive CD4^+^ T cells into Th17 effector cells is primarily driven by TGF-β and IL-6, with IL-23 playing a critical role in their survival and expansion [101,102]. Moreover, IL-23 induces the expression of RORγt in Th17 cells, which subsequently promotes the expression of IL-23R and IL-17 [103]. In several experimental psoriasis models, IL-23 acts as a pivotal upstream regulator, bridging innate and adaptive immune responses by promoting the differentiation and maintenance of Th17 cells [104]. When administered to healthy mouse skin, IL-23 triggered the development of psoriasis-like lesions, characterized by epidermal hyperplasia, inflammatory cell infiltration, and the upregulation of several pro-inflammatory cytokines [105,106]. In contrast, IL-23p19^−/−^ mice were protected from imiquimod-induced psoriasis, further emphasizing the critical role of IL-23 [105]. When IL-23 binds to the IL-23R complex, it activates intracellular signaling pathways, including the STAT3 pathway, leading to the production of effector cytokines such as IL-17A, IL-17F, and IL-22. Among these, IL-22 plays a crucial role in promoting keratinocyte hyperproliferation and impairing normal differentiation, resulting in the epidermal thickening (acanthosis) and abnormal cornification (parakeratosis) observed in psoriatic lesions [107]. Furthermore, IL-23 promotes the expansion of other IL-17-producing cells, such as CD8^+^ T lymphocytes, ILC3s, and γδ T cells, thereby amplifying the inflammatory response [108]. The extreme production of IL-23 in psoriatic skin establishes a self-perpetuating inflammatory loop, in which activated keratinocytes release chemokines that attract additional immune cells, thereby sustaining the inflammatory cascade [109]. In the context of IBD, IL-23 plays a crucial role in promoting the differentiation and expansion of Th17 cells, which are key drivers of chronic intestinal inflammation [110]. In IL-10^−/−^ mice, a preclinical model of IBD, IL-23 has been shown to be vital for the development of chronic intestinal inflammation [111]. IL-23 stimulates the production of pro-inflammatory cytokines such as IL-17A, IL-17F, and IL-22 by Th17 cells and other IL-23R-expressing populations, including γδ T cells and ILCs [112]. These cytokines, in turn, promote neutrophil recruitment, impair epithelial barrier function, and perpetuate the inflammatory cascade [113]. The significance of IL-23 in IBD pathogenesis is supported by genome-wide association studies linking IL23R gene polymorphisms to increased susceptibility to IBD [114,115]. The association has been confirmed in several cohorts which showed a significant association with Crohn’s disease (CD; a subtype of IBD), with the strongest signal for the coding variant Arg381Gln [115]. In a model of ulcerative colitis (UC; another subclass of IBD), the administration of recombinant IL-23 accelerated disease onset and exacerbated severity, highlighting its pro-inflammatory role [116].

Beyond its effects on immune cell dynamics, IL-23 has been implicated in the direct modulation of nociceptive pathways through its influence on peripheral and central sensitization mechanisms [117,118]. Studies have suggested that IL-23 may enhance pain signaling by acting on nociceptive neurons or by indirectly modulating the inflammatory milieu within affected tissues, leading to the highly increased excitability of pain-transmitting pathways [95]. A fundamental element in the pro-nociceptive effects of IL-23 is the action of cyclooxygenases (COX-1 and COX-2). These enzymes play a central role in converting arachidonic acid into bioactive eicosanoids, including prostaglandins (PGs) and thromboxanes, which are established mediators of inflammation and pain [94,95,119]. This interdependence of IL-23 and COX-derived metabolites suggests the existence of multiple converging signaling pathways that synergistically potentiate peripheral and central sensitization mechanisms. The role of COX-generated eicosanoids in nociception is well documented, with prostaglandins (especially PGE_2_) being key contributors to nociceptor sensitization by reducing their activation threshold and elevating pain perception [120,121].

Studies have indicated that IL-23 plays a key role in the development of arthritis pain, with its activity potentially linked to COX function [93,94,95]. In some experimental models, the injection of IL-23 into the plantar region induced inflammatory pain, which was alleviated by indomethacin [94]. This suggests that IL-23 may contribute to pain development in RA through pathways involving COX-mediated prostaglandin synthesis. On the other hand, although a direct relationship between IL-23 and cyclooxygenases has not yet been established, a notable increase in COX-2 expression has been observed in patients with MS, especially in regions of recent demyelination [122]. In the context of IBD, the interaction between IL-23 and COX may exacerbate inflammation and contribute to pain symptoms. Recent studies have shown that novel 1-H phenyl benzimidazole derivatives, which target the IL-23-mediated inflammatory pathway, can reduce COX-2 activity [123]. With respect to psoriasis, there is no connection between IL-23 and COX-2; however, a recent study marked the relevance of COX-2 in the activity of macrophages [124].

IL-23 has also been implicated in the activation of the lipoxygenase (LOX) pathway, another major branch of arachidonic acid metabolism, which leads to the biosynthesis of leukotrienes [125]. Among these lipid mediators, leukotriene B_4_ (LTB_4_) has garnered particular attention due to its ability to activate and recruit innate immune cells, such as neutrophils and macrophages, to sites of inflammation [126,127]. The role of LTB_4_ in pain extends beyond its pro-inflammatory effects, as it has been shown to directly modulate pain signaling through its interactions with nociceptors. Specifically, LTB_4_ acts on leukotriene receptors (BLT1 and BLT2), which are expressed on nociceptors and immune cells, leading to the activation of several intracellular signaling cascades that worsen pain hypersensitivity [128,129]. This mechanism is important in chronic inflammatory conditions, such as RA, where IL-23-mediated immune activation contributes to joint destruction and persistent pain [130]. Research has elucidated that IL-23 enhances the production of LTB_4_ by stimulating the activity of innate immune cells within the synovial microenvironment, thus evoking a self-reinforcing cycle of inflammation [130]. The LOX pathway, especially 5-lipoxygenase (5-LO), plays a role in neuroinflammation and axonal injury in MS [131]. The increased expression of 5-LO has been observed in MS lesions, and elevated leukotriene levels have been detected in the cerebrospinal fluid of MS patients [132]. The inhibition of 5-LO has significantly reduced microglial activation, decreased IL-6 levels, and alleviated axonal injury and motor impairments in several animal studies [133,134,135]. Regarding IL-23, dendritic cells derived from MS patients release higher quantities of IL-23 and exhibit elevated levels of IL-23p19 mRNA, resulting in increased IL-17 production by Th17 cells [136]. On the other hand, some studies have demonstrated the elevated expression of 5-LO pathway proteins in colonic biopsies from patients with IBD, particularly ulcerative colitis (UC). This upregulation correlates with the increased infiltration of some inflammatory cells, such as neutrophils, macrophages, and T cells [137,138,139]. Finally, 12R-lipoxygenase (12R-LOX) contributes to the development of psoriatic lesions. Studies have shown that 12R-LOX is pathologically overexpressed in psoriasis, leading to the strong accumulation of 12R-hydroxyeicosatetraenoic acid (12R-HETE) in the skin [140,141]. The presence of 12R-LOX has been implicated in the proliferative properties of psoriatic skin disorders [142].

**Figure 4:**
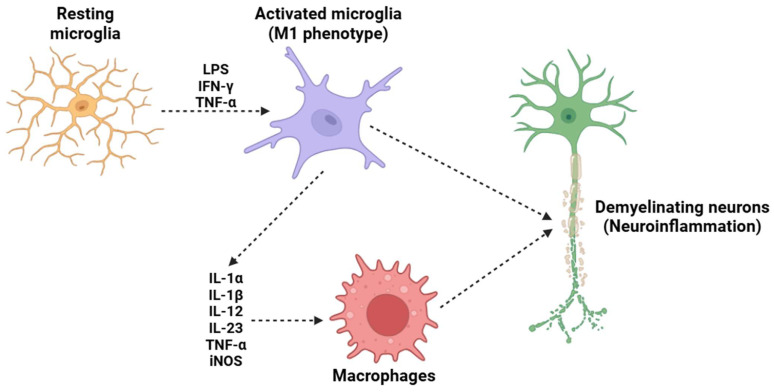
A potential mechanism underlying the demyelination process in MS is the activation of microglia. Under resting conditions, microglia maintain a resting state; however, in the presence of LPS, IFN-γ, and TNF-α, they adopt a pro-inflammatory M1 phenotype. This phenotypic change induces the release of several pro-inflammatory cytokines (such as IL-1α, IL-1β, IL-12, IL-23, and TNF-α) and the activation of iNOS. This subsequently attracts circulating macrophages, which cross the BBB and migrate into the CNS. The combined effects of resident microglia and circulating macrophages trigger a neuroinflammatory process that ultimately results in the destruction of the neuronal myelin sheath, a critical mechanism in the pathogenesis of MS. Abbreviations: LPS (lipopolysaccharide), IFN-γ (interferon gamma), TNF-α (tumor necrosis factor alpha), IL-1α (interleukin 1 alpha), IL-1β (interleukin 1 beta), IL-12 (interleukin 12), IL-23 (iterleukin 23), and iNOS (inducible nitric oxide synthase).

Monocytes/macrophages/microglia (Figure 4) are crucial cellular mediators of IL-23-induced nociceptive effects, serving as crucial cellular intermediaries in the inflammatory pain cascade. Experimental strategies involving monocyte and macrophage depletion have provided strong evidence supporting the role of these immune cells in IL-23-induced mechanical hypersensitivity [143,144,145,146]. Some research employing clodronate liposomes (used in a well-established method for the depletion of phagocytic monocytes and macrophages) has demonstrated that the absence of these immune cells effectively prevents IL-23-induced mechanical allodynia and hyperalgesia [147]. These findings highlight that the activity of macrophages is indispensable for the transmission and amplification of IL-23-mediated pain signals. Upon exposure to IL-23, macrophages shift to a pro-inflammatory state, leading to the release of IL-17, a pro-inflammatory cytokine with well-documented effects on immune regulation and pain modulation [148]. IL-17, rather than IL-23 itself, is responsible for the activation of nociceptors [149]. This process occurs through the binding of IL-17 to its cognate receptor, IL-17RA, which is expressed on peripheral sensory neurons, including those within the DRG [150,151]. The strong activation of these nociceptive neurons by IL-17 triggers intracellular signaling cascades, including the activation of MAPKs, NF-κB, and TRPs, such as TRPV1 and TRPA1 [144,152]. Furthermore, the macrophage-derived IL-17 signaling pathway not only contributes to acute nociceptive hypersensitivity but also plays a crucial role in the maintenance and persistence of chronic inflammatory pain states. IL-17 is known to enhance the production of additional pro-inflammatory mediators, including TNF-α and prostaglandins (such as PGE_2_), all of which sensitize nociceptors and exacerbate pain signaling [153,154,155]. This reinforces the concept that IL-23-induced pain is not solely a transient inflammatory event but rather part of a broader neuroimmune interaction that sustains long-term pain sensitization.

In conclusion, the interplay between IL-23, macrophages, and IL-17 represents a crucial nexus in the development of inflammatory pain. The indirect activation of nociceptors via macrophage-derived IL-17 highlights the broader concept of neuroimmune crosstalk in pain pathophysiology. Targeting this axis offers promising therapeutic avenues for mitigating inflammatory pain conditions. Further research into these pathways will be crucial for refining targeted interventions and improving the clinical management of chronic inflammatory pain syndromes.

## 4. Therapeutic Targeting of IL-23 in Inflammatory Diseases

Due to its essential role, therapeutic strategies have been developed to directly target IL-23, aiming to interrupt its signaling pathway. Immunotherapy is a treatment approach focused on boosting the immune system’s ability to fight various diseases, including cancer [156], neuropathies [157], and autoimmune disorders [158]. This approach has gained as a promising alternative to traditional therapies, offering the benefit of fewer side effects [159]. Monoclonal antibody-based immunotherapies have proven effective in managing the progression of numerous chronic conditions by modulating the interactions between immune cells and the PNS/CNS, leading to substantial relief from the painful symptoms associated with these diseases [160].

Table 1 summarizes the most relevant immunotherapeutic treatments targeting IL-23, emphasizing its effectiveness in preventing the onset of various inflammatory diseases and mitigating the severity of pain associated with these conditions.

## 5. Conclusions

This review offers an analysis of the involvement of the IL-23 cytokine in the intricate mechanisms underlying inflammatory pain. As a pro-inflammatory cytokine, IL-23 influences immune cell regulation and the activation of some signaling pathways, contributing to pain sensitization. IL-23 not only intensifies the severity of inflammatory pain but also contributes to its persistence and chronicity, highlighting its potential as a therapeutic target for interventions aimed at mitigating chronic pain and enhancing patient outcomes.

The growing body of evidence supports the notion that modulating IL-23 might offer a promising therapeutic strategy for relieving inflammatory pain. Several studies suggest that therapies targeting IL-23 show potential in relieving pain symptoms. Given the complexity of inflammatory pain and the multifaceted involvement of IL-23 in its pathophysiology, further research is necessary to fully elucidate the mechanisms underlying its biological effects. Additional studies are required to confirm these early findings and assess the long-term efficacy, safety, and side effects of monoclonal anti-IL-23 therapies. Focusing on IL-23R rather than IL-23 itself offers a promising therapeutic approach. Orally bioavailable peptides that specifically target IL-23R may provide superior tissue penetration compared to antibodies, potentially enhancing treatment outcomes for inflammatory diseases [180,181].

In conclusion, this article aims to offer valuable insights to guide the development of innovative and more effective treatment strategies for inflammatory pain. Such advancements could ultimately enhance patient care and significantly improve the quality of life for individuals suffering from chronic inflammatory pain.

## Figures and Tables

**Table 1:** A list of anti-IL-23 monoclonal antibodies used in clinical trials. Abbreviations: PASI75 (psoriasis area and severity index—75% reduction), ACR20 (American College of Rheumatology—20% improvement), BASDAI20 (bath ankylosing spondylitis disease activity index—20% improvement), BASDAI50 (bath ankylosing spondylitis disease activity index—50% improvement), BASDAI70 (bath ankylosing spondylitis disease activity index—70% improvement), PASI90 (psoriasis area and severity index—90% reduction), PASI100 (psoriasis area and severity index—100% reduction), SF (stool frequency), RB (rectal bleeding), BU (bowel urgency), AP (abdominal pain), and CDAI (Crohn’s disease activity index).

Inflammatory Disease	Antibody Employed	Effects	References
Rheumatoid arthritis	Ustekinumab (anti-IL-12/23p40)	Treatment with ustekinumab (90 mg at weeks 0 and 4 and every 8 weeks or 90 mg at weeks 0 and 4 and every 12 weeks) and guselkumab (50 mg at weeks 0 and 4 and every 8 weeks or 200 mg at weeks 0 and 4 and every 8 weeks until week 28) resulted in modest improvements in the signs and symptoms of rheumatoid arthritis, although no significant differences were observed (clinical trial No. NCT01645280).	[161]
	Guselkumab (anti-IL-23p19)		
Psoriasis	Ustekinumab (anti-IL-12/23p40)	Ustekinumab demonstrated a high degree of efficacy in the treatment of psoriasis in four Phase III clinical trials (No. NCT00267969, No. NCT00307437, No. NCT01009086, and No. NCT01077362). The efficacy was as follows: (1) No. NCT00267969 (67.1% of patients receiving 45 mg of ustekinumab at weeks 0 and 4 and then every 12 weeks, 66.4% receiving 90 mg of ustekinumab at weeks 0 and 4 and then every 12 weeks, and 3.1% receiving a placebo achieved PASI75 after 12 weeks of treatment); (2) No. NCT00307437 (66.7% of patients receiving 45 mg of ustekinumab at weeks 0 and 4 and then every 12 weeks, 75.7% receiving 90 mg of ustekinumab at weeks 0 and 4 and then every 12 weeks, and 3.7% receiving a placebo achieved PASI75 after 12 weeks of treatment); (3) No. NCT01009086 (42.4% of patients in the group receiving 45 mg of ustekinumab at weeks 0 and 4 and then every 12 weeks, 49.5% in the group receiving 90 mg at weeks 0 and 4 and then every 12 weeks, and 22.8% in the placebo group achieved ACR20 at week 24); (4) NCT01077362 (at week 24, patients receiving 45 or 90 mg of ustekinumab —administered at weeks 0 and 4, and subsequently maintained every 12 weeks—achieved BASDAI20/50/70 response rates of 54.8%/29.3%/15.3%, compared to 32.9%/11.4%/0% in the placebo group).	[162,163,164,165]
	Briakinumab (anti-IL-12/23p40)	In a Phase II dose-ranging psoriasis trial (No. NCT00292396), briakinumab exhibited a significant enhancement in PASI75 reduction at week 12 across all treatment groups in comparison with the placebo: (1) 200 mg once (63%); (2) 100 mg every other week for 12 weeks (93%); (3) 200 mg weekly for 4 weeks (90%); (4) 200 mg every other week for 12 weeks (93%); (5) 200 mg weekly for 12 weeks (90%); (6) placebo (3%).	[166]
	Tildrakizumab (anti-IL-23p19)	In the 28-week stage of the study, administered tildrakizumab (200 mg, every 12 weeks) demonstrated the following efficacy rates: 78% of patients achieved PASI75, 58% achieved PASI90, and 29% achieved PASI100 (No. NCT01722331 and No. NCT01729754).	[167]
	Guselkumab (anti-IL-23p19)	Guselkumab (100 mg; weeks 0 and 4, then every 8 weeks) was demonstrated to be an effective treatment for psoriasis in two Phase III trials (No. NCT02207231 and No. NCT02207244). At week 16, 73.3% of patients achieved PASI90, while 37.4% achieved PASI100.	[168]
	Risankizumab (anti-IL-23p19)	At week 16, risankizumab (150 mg at weeks 0 and 4) achieved PASI90 in 75% of patients (clinical trial No. NCT02207231).	[169]
Inflammatory bowel disease (UC)	Risankizumab (anti-IL-23p19)	In an induction trial (No. NCT03398148; 1200 mg at weeks 0, 4, and 8), clinical remission rates were observed to be 20.3% for risankizumab at week 12. In the maintenance trial (No. NCT03398135; 180 or 360 mg every 8 weeks for 52 weeks), remission rates were found to be 40.2% for risankizumab at week 52.	[170]
	Guselkumab (anti-IL-23p19)	In a Phase IIb trial (No. NCT04033445; 200 or 400 mg at weeks 0, 4, and 8), the clinical response rate at week 12 was found to be significantly higher with guselkumab (61.4%) than with a placebo (27.6%).	[171]
	Mirikizumab (anti-IL-23p19)	In clinical trials (No. NCT03518086 (300 mg at weeks 0, 4, and 8 for 12 weeks) and No. NCT03524092 (200 mg every 4 weeks, beginning at week 12 and continuing until week 40), mirikizumab demonstrated superior efficacy in comparison to a placebo. At week 2, greater reductions in SF, RB, BU, and fatigue were achieved with mirikizumab. At week 4, a higher rate of AP improvement was observed. At week 12, a greater number of patients had achieved symptomatic remission or meaningful improvement in RB, SF, and BU. Symptom control was maintained consistently through 52 weeks.	[172]
	Briakinumab (anti-IL-12/23p40)	In a clinical trial (No. NCT00562887 (induction phase: placebo or 200, 400, or 700 mg of briakinumab at weeks 0, 4, and 8; maintenance phase: placebo or 200 or 700 mg of briakinumab at weeks 12, 16, and 20), the primary endpoint of achieving clinical remission at week 6 was not reached. However, patients treated with briakinumab showed higher remission and response rates at weeks 6, 12, and 24 (approximately 60% vs. 20% in the placebo group).	[173]
Inflammatory bowel disease (CD)	Risankizumab (anti-IL-23p19)	In two clinical trials (No. NCT03105128 and No. NCT03104413), patients were assigned to 600 mg or 1200 mg of risankizumab or a placebo at weeks 0, 4, and 8. In the No. NCT03105128 clinical trial, 600 mg of risankizumab demonstrated a CDAI clinical remission rate of 45%, with both 600 mg and 1200 mg also showing higher rates of clinical remission for stool frequency and abdominal pain score, as well as an improved endoscopic response compared to placebo. A similar outcome was observed in the No. NCT03104413 clinical trial, with risankizumab achieving superior clinical and endoscopic outcomes in comparison to the placebo.	[174]
	Risankizumab (anti-IL-23p19)	In a Phase III clinical trial (No. NCT03105102), patients were randomized to receive 180 mg or 360 mg of risankizumab or a placebo every 8 weeks. The 360 mg dose demonstrated higher rates of CDAI clinical remission vs. the placebo group (52% vs. 41%), as well as improvements in stool frequency and abdominal pain score remission (52% vs. 40%) and endoscopic response (47% vs. 22%).	[175]
	Guselkumab (anti-IL-23p19)	In a clinical trial (No. NCT03466411), guselkumab (intravenous, 200 mg, 600 mg, or 1200 mg at weeks 0, 4, and 8; intravenous, 6 mg/kg at week 0; 90 mg, subcutaneous, at week 8) demonstrated higher rates of CDAI clinical remission at week 48 (57.4%, 55.6%, and 45.9% vs. 16.4%). Furthermore, the endoscopic response rate was found to be higher in the guselkumab groups.	[176,177]
	Mirikizumab (anti-IL-23p19)	In a clinical trial (No. NCT02891226), mirikizumab (200, 600, or 1000 mg of mirikizumab, administered every 4 weeks) demonstrated a significantly higher endoscopic response rate compared to the placebo in all treatment groups at week 12, with the highest response rate (43.8%) in the 1000 mg group.	[178]
	Mirikizumab (anti-IL-23p19)	In a clinical trial (No. NCT03926130), mirikizumab (900 mg intravenously administered at weeks 0, 4, and 8; then, 300 mg subcutaneously administered every 4 weeks from weeks 12 to 52) demonstrated significant superiority over the placebo in both primary endpoints: the endoscopic response (38% vs. 9%) and CDAI clinical remission (45% vs. 20%).	[179]

## Data Availability

Not applicable. No new data were generated.

## Abbreviations

The following abbreviations are used in this manuscript:

12R-LOX	12R-lipoxygenase
12R-HETE	12R-hydroxyeicosatetraenoic acid
5-LO	5-lipoxygenase
ACR20	American College of Rheumatology (20% improvement)
AP	Abdominal pain
APC	Antigen-presenting cell
BASDAI20	Bath ankylosing spondylitis disease activity index (20% improvement)
BASDAI50	Bath ankylosing spondylitis disease activity index (50% improvement)
BASDAI70	Bath ankylosing spondylitis disease activity index (70% improvement)
BBB	Blood–brain barrier
BLT1	Leukotriene B4 receptor 1
BLT2	Leukotriene B4 receptor 2
BU	Bowel urgency
CCL20	C-C motif chemokine ligand 20
CCR6	C-C chemokine receptor type 6
CD	Crohn’s disease
CD4	Cluster of differentiation 4
CD8	Cluster of differentiation 8
CDAI	Crohn’s disease activity index
CNS	Central nervous system
COX-1	Cyclooxygenase 1
COX-2	Cyclooxygenase 2
CTL	Cytotoxic T cells
CXCL1	C-X-C motif chemokine ligand 1
CXCL9	C-X-C motif chemokine ligand 9
DAMP	Damage-associated molecular pattern
DCs	Dendritic cells
dDCs	Dermal dendritic cells
DRG	Dorsal root ganglion
GALT	Gut-associated lymphoid tissue
GM-CSF	Granulocyte-macrophage colony-stimulating factor
HIF-1α	Hypoxia-inducible factor 1 alpha
IBS	Inflammatory bowel syndrome
IFN-γ	Interferon gamma
IKK	IκB kinase
IL-10	Interleukin 10
IL-12	Interleukin 12
IL-12p40	Interleukin 12 p40 subunit
IL-12Rβ1	Interleukin 12 receptor β1
IL-17	Interleukin 17
IL-17A	Interleukin 17A
IL-17F	Interleukin 17F
IL-1α	Interleukin 1 alpha
IL-1β	Interleukin 1 beta
IL-23	Interleukin 23
IL-23p19	Interleukin 23 p19 subunit
IL-23R	Interleukin 23 receptor
IL-27	Interleukin 27
IL-35	Interleukin 35
IL-6	Interleukin 6
ILC	Innate lymphoid cell
ILC3	Innate lymphoid cell 3
iNOS	Inducible nitric oxide synthase
JAK	Janus kinase
JAK2	Janus kinase 2
JAK-STAT	Janus kinase-signal transducer and activator of transcription pathway
LOX	Lipoxygenase
LPS	Lipopolysaccharide
LTB4	Leukotriene B4
MAIT	Mucosal-associated invariant T cell
MAPK	Mitogen-activated protein kinase
MDSCs	Myeloid-derived suppressor cells
MS	Multiple sclerosis
NF-κB	Nuclear factor kappa-light-chain-enhancer of activated B cells
NK	Natural killer cell
NKT	Natural killer T cell
NLR	NOD-like receptor
NOD	Nucleotide-binding oligomerization domain
p19	p19 subunit
p40	p40 subunit
p50	p50 subunit
p65	p65 subunit
PAMP	Pathogen-associated molecular pattern
PASI75	Psoriasis area and severity index (75% reduction)
PASI90	Psoriasis area and severity index (90% reduction)
PASI100	Psoriasis area and severity index (100% reduction)
PG	Prostaglandin
PGE_2_	Prostaglandin E2
PNS	Peripheral nervous system
PRR	Pattern recognition receptor
RA	Rheumatoid arthritis
RB	Rectal bleeding
RORγt	Retinoic acid receptor-related orphan receptor gamma t
SF	Stool frequency
SPM	Specialized pro-resolving mediator
STAT3	Signal transducer and activator of transcription 3
TGF-β	Transforming growth factor beta
Th	T helper cell
Th1	T helper 1 cell
Th17	T helper 17 cell
Th2	T helper 2 cell
TLR	Toll-like receptor
TME	Tumor microenvironment
TNF-α	Tumor necrosis factor alpha
TRP	Transient receptor potential
TRPA1	Transient receptor potential ankyrin 1
TRPV1	Transient receptor potential vanilloid 1
TYK2	Tyrosine kinase 2
UC	Ulcerative colitis
VEGF	Vascular endothelial growth factor
γδ T	Gamma delta T cell
